# Quantitative Proteomics Reveal Peroxiredoxin Perturbation Upon Persistent Lymphocytic Choriomeningitis Virus Infection in Human Cells

**DOI:** 10.3389/fmicb.2019.02438

**Published:** 2019-10-25

**Authors:** Martin Benej, Maksym Danchenko, Ingrid Oveckova, Filip Cervenak, Lubomir Tomaska, Katarina Grossmannova, Katarina Polcicova, Tereza Golias, Jana Tomaskova

**Affiliations:** ^1^Biomedical Research Center, Institute of Virology, Slovak Academy of Sciences, Bratislava, Slovakia; ^2^Faculty of Natural Sciences, Department of Genetics, Comenius University in Bratislava, Bratislava, Slovakia

**Keywords:** LCMV, arenavirus, host response, proteomics, peroxiredoxin, ROS, redox signaling, telomeres

## Abstract

Experimental data indicate that during persistent infection, lymphocytic choriomeningitis virus (LCMV) may both directly or indirectly modulate regulatory cellular processes and alter cellular functions that are not critical for survival, but are essential for cell homeostasis. In order to shed more light on these processes, two-dimensional differential in-gel electrophoresis (2D-DIGE) and MALDI-TOF tandem mass spectrometry were used to determine the proteome response of the HeLa cell line to persistent LCMV infection. Quantitative analysis revealed 24 differentially abundant proteins. Functional analysis showed that LCMV-responsive proteins were primarily involved in metabolism, stress, and the defense response. Among identified proteins, we discovered significant changes for peroxiredoxins, a family of antioxidant enzymes. Decreased amount of these antioxidant proteins correlated with elevation of reactive oxygen species (ROS) in infected cells. Increased levels of ROS were accompanied by changes in the pattern of telomere restriction fragments (TRFs) in infected cells and mediated activation of hypoxia-inducible transcription factor-1 (HIF-1) and phosphatidylinositol 3-kinase (PI3K)/Akt signaling pathways. Moreover, treatment with antioxidants resulted in reduced levels of viral nucleoprotein, indicating a connection between ROS-dependent signaling and viral replication.

## Introduction

Persistent viral infections are currently one of the most important global health problems in the world. Understanding how viral persistence is initiated and maintained, and the pathological consequences of ongoing virus replication, is therefore of high importance.

The prototypic mammarenavirus, lymphocytic choriomeningitis virus (LCMV), provides a suitable model system for investigation of the mechanisms of persistent viral infection, virus-host interactions, and pathogenesis. Studies using this viral model have resulted in significant progress in virology and immunology that is applicable to other human microbial and viral infections (Oldstone, 2002; Zinkernagel, 2002). In addition, LCMV is a neglected human pathogen of clinical significance that may lead to severe disease and consequences after prenatal infection (Barton and Mets, 1999; Mets et al., 2000; Barton et al., 2002), and life-threatening conditions in immunosuppressed individuals (Fischer et al., 2006; Palacios et al., 2008; Macneil et al., 2011). Moreover, several mammarenaviruses, such as the Lassa, Junin, and Machupo viruses, are associated with severe hemorrhagic fever disease with significant morbidity and mortality in humans, representing serious public health concerns in their endemic regions (Geisbert and Jahrling, 2004; Buchmeier et al., 2007). Currently, supportive care and ribavirin (nucleoside analogue) are the only treatment options available (Damonte and Coto, 2002; Parker, 2005). Therefore, considerable interest focuses on the development of therapeutic strategies against mammarenavirus infection and disease. Understanding how viruses and the hosts interact and how their interactions change over time would help in the rational design of targeting compounds.

Mammarenaviruses are enveloped viruses with a bisegmented negative-strand RNA genome and a non-lytic life cycle restricted to the cell cytoplasm. The S segment of the genome encodes the nucleoprotein (NP) that encapsidates viral RNA and the glycoprotein precursor (GPC). After post-translational modification, GPC yields GP1 and GP2 that mediate virus entry. The L segment encodes the viral RNA-dependent RNA polymerase (L) responsible for viral RNA synthesis, and the zinc-binding protein (Z) important for viral budding (Buchmeier et al., 2007). Mammarenaviruses are able to establish persistent infection in their natural hosts and in a number of mammalian cells *in vitro*. During persistence, mammarenaviruses continue to replicate and express viral proteins. At the same time, they interfere with normal host homeostasis, resulting in possible disease without the destruction of the infected cell. Additionally, they actively suppress the host’s immune response in order to avoid recognition and to allow the spread of infection (Oldstone, 2006). Although research on LCMV has led to many insights into viral persistence, the answers to several crucial questions related to the principles, by which persistence is initiated and maintained, are still lacking.

A highly suitable tool to study virus-host cell interactions is proteomics, since viral infection fundamentally affects host cell proteins. It does so at many functional levels, such as affecting the cell signaling pathways, cytokine and growth factor production, apoptosis, phagocytosis, protein degradation, and cytoskeletal rearrangement (Coiras et al., 2008). So far, several large-scale analyses of host proteome (Bowick et al., 2006, 2009, 2010), kinome (Bowick et al., 2007), and transcriptome (Djavani et al., 2007, 2009; Muller et al., 2007) have been performed to study the consequences of an acute mammarenavirus infection. Moreover, a number of studies employed the proteomic approach to identify interacting partners of mammarenavirus proteins in human cells (Khamina et al., 2017; King et al., 2017; Iwasaki et al., 2018; Loureiro et al., 2018; Ziegler et al., 2018). On the other hand, none of them has focused on the host cellular response during persistent infection.

Herein, we analyzed proteomic changes in a human cell line HeLa upon persistent infection with the MX isolate of LCMV using quantitative gel-based proteomics. Our data reveal significant changes in the proteins associated with metabolism, antioxidant activity, protein folding, RNA binding, and immune response. Many of the differentially abundant proteins have not been reported previously as virus-responsive upon LCMV infection. Thus, these proteins represent potential targets for further in-depth investigations. Understanding their roles in persistent LCMV infection would reveal more about the complexity and dynamics of interactions between virus and host cells, and would benefit antiviral research. This study thus offers useful basis for further research into the function of human proteins in persistent infection.

## Materials and Methods

### Biological Material

Human cervical carcinoma cells (HeLa), human alveolar adenocarcinoma cells (A549) (ATCC CCL-185), and baby hamster kidney fibroblast cells (BHK-21) (ATCC CCL-10), were grown in Dulbecco’s modified Eagle medium (DMEM) containing 10% fetal calf serum (FCS) supplemented with 40 μg/ml gentamicin in a humidified atmosphere with 5% CO_2_ at 37°C. LCMV MX isolate was continuously propagated in persistently infected HeLa cells (designated HeLa-MX). The MX isolate represents a persistent form of the virus capable of propagation through cell-cell contacts without releasing infectious virions (Gibadulinova et al., 1998; Reiserova et al., 1999; Tomaskova et al., 2008). A549 cells were infected with MX isolate via cell-free extract from persistently infected BHK-21 cells (BHK-MX) as described in Laposova et al. (2017) and subsequently passaged several times to allow virus spread.

### Protein Extraction

Whole-proteome samples for 2D-DIGE analysis were prepared according to standard protocol. Briefly, the cells were washed with ice-cold PBS, and 600 μl of lysis buffer (150 mM NaCl; 1 M Tris, pH 7.5; 1% Triton X-100; and 0.1% SDS) were added to the culture, followed by 20 min incubation on ice. The lysed cells were scraped off, transferred to tubes, passaged through an insulin syringe and centrifuged at 17,000 × *g* for 15 min at 4°C. Next, nine volumes of precipitation solution (acetone:methanol 9:1) were added, and the resulting solutions were incubated overnight at −20°C. The samples were then centrifuged at 9000 × *g* for 30 min at 4°C. Obtained pellets were air-dried and resuspended in 300 μl of UTC solution (7M Urea, 2M Thiourea, 4% CHAPS). Final protein concentrations were determined using the Bradford method (Bio-Rad).

### Preparative 2D Gel

For the preparative gel, 100 μg of each sample were mixed with the UTC solution to the final volume of 225 μl. An equal volume of loading/reswelling buffer [65 mM dithiothreitol (DTT); 2% (v/v) immobilized pH gradient (IPG) buffer, pH 3–10 NL (GE Healthcare), 2.8% DeStreak reagent (GE Healthcare); adjusted with UTC solution to the final volume of 500 μl] was added to the mixture. Eighteen centimeters IPG strip with non-linear 3–10 pH range (GE Healthcare) was passively reswelled in 450 μl of the sample mixture overnight. Isoelectric focusing was performed using the Multiphor II unit (GE Healthcare), with the following 6-step protocol: (1) 100 V, 2 h; (2) 150 V, 2 h; (3) 600 V, 2 h; (4) 1000 V, 1 h; (5) 5000 V, 2 h; and (6) 5000 V, 16 h. The current limit was set to 2 mA, with a ramping voltage gradient between the steps.

After separation in the 1st dimension, the strip was equilibrated in SDS Equilibration solution (6M Urea; 2% SDS; 20% glycerol; 37.5 mM Tris–HCl pH 8.8) containing 0.5% (w/v) DTT for 15 min; followed by 15 min incubation with SDS Equilibration solution in the presence of 4.5% iodoacetamide (IAA). The equilibrated strip was placed on top of a 12% polyacrylamide gel cast between low-fluorescence glass plates (the backing plate being pretreated with Bind Silane solution; 4 ml 96% ethanol, 1.8 ml mQ water, 0.2 ml acetic acid and 10 μl of Bind Silane, 1 h incubation at room temperature) and fixed by adding 0.5% agarose gel dissolved in mQ water with a trace of bromophenol blue.

Separation in the 2nd dimension was carried out using the Protean XL (Bio-Rad) chamber. The resulting glass-backed 2D gel was incubated 2 × 30 min in Fixing Solution (500 ml ethanol, 70 ml acetic acid, 430 ml mQ water), the solution being replaced between individual fixing steps. Total proteins were stained by overnight incubation with SYPRO Ruby Protein Gel Stain (Molecular Probes) in the dark. The gel was washed 2 × 30 min in Wash Solution (100 ml methanol, 70 ml acetic acid, 830 ml mQ water) and 2 × 10 min in mQ water prior to scanning. Resulting 2D map of the proteome was visualized by the PharosFX Molecular Imager (Bio-Rad), using 50 μm resolution and appropriate laser/filter wavelengths.

### Sample Labeling and 2D-DIGE Analysis

In the first step, sample pH was adjusted to fit the range of 8.0–9.0 using 1 M Tris. Fluorescent dyes Cy2, Cy3, and Cy5 were used for minimal sample labeling (GE Healthcare). Fifty micrograms of each sample were labeled with 400 pmol of either Cy3 or Cy5, in biological triplicates, using the dye-swap technique between individual replicate pairs. For the internal standard, 25 μg of each sample replicate was mixed with 400 pmol of Cy2 per sample. After 30 min of on-ice incubation in the dark, the labeling reaction was stopped by adding 1/10 volume of 10 mM L-lysine to the samples. After a short spin, the labeled mixtures were incubated on ice for 10 min in the dark. Subsequently, the samples for each replicate gel were mixed in the following manner Cy2:Cy3:Cy5 1:1:1, adjusted with UTC solution to the final volume of 225 μl and mixed with an equal volume of the loading/reswelling buffer. IPG strips with non-linear 3–10 pH range were passively rehydrated in 450 μl of the sample mixtures overnight in the dark.

The 2D separation protocol was identical to that of the preparative gel with three exceptions – (i) 12% polyacrylamide gel was cast between two low-fluorescence glass plates, not pre-treated with Bind-Silan; (ii) the proteins in the analytical gels were not fixed, but directly scanned (iii) particular emphasis was put on working in the dark. Obtained 2D gels were immediately scanned between two low-fluorescence glass plates using the PharosFX Molecular Imager at 50 μm resolution and at appropriate excitation/emission wavelengths corresponding to Cy2, Cy3, and Cy5 individual channels.

### Quantitative Gel Analysis

Quantitative gel analysis was carried out using DeCyder 7.2 software package (GE Healthcare). For those proteins discovered to be different in mock-infected and LCMV-infected samples, analysis of significance was conducted using the Student’s *T*-test. Only significantly different protein spots (*P* < 0.05), with an at least 1.5-fold abundance difference (ratio of mock versus infected sample mean normalized spot signals) were regarded as up- or down-regulated. These protein spots of interest were chosen for mass spectrometry detection. Target spots of interest were excised from the preparative gel using EXQuest Spot Cutter (Bio-Rad) and destained. After reduction and alkylation, the gel plugs were digested overnight with sequencing grade modified trypsin (Promega). The digested peptides were extracted with 60 μl of 50% acetonitrile (Merck) containing 0.1% trifluoroacetic acid (TFA) (Merck).

### Protein Identification by Tandem Mass Spectrometry

Samples with digested peptides were evaporated to 20 μl in Concentrator plus (Eppendorf) and purified using μ-C18 ZipTips (Merck Millipore). Subsequently, 1 μl of peptide mixtures and 1 μl of 0.7 mg/ml CHCA matrix in 85% acetonitrile, 0.1% TFA, and 1 mM ammonium phosphate were pipetted onto 800 μm AnchorChip MALDI target (Bruker).

Data were acquired on ultrafleXtreme TOF mass spectrometer (Bruker) operated by flexControl 3.3 (Bruker) in positive reflector mode. For each position on the target 4000 laser shots were summed in 700–3500 m/z range. Next, we selected the 25 most intense precursor ions per sample for fragmentation with signal-to-noise threshold 15. Tandem mass spectra were recorded by accumulation of 3000 shots in LIFT mode using LID (laser-induced dissociation) mechanism. Laser power was boosted by 50% without collision gas, and the detector voltage was increased by 80%.

Monoisotopic peak lists were generated in flexAnalysis 3.3 (Bruker) by SNAP algorithm. Precursor spectra with signal-to-noise less than ten were removed, and the remaining masses were externally recalibrated. Fragment spectra were smoothed using Savitzky–Golay algorithm (three cycles with 0.15 m/z width), the baseline was subtracted with TopHat algorithm, and filtering was done on signal-to-noise level five.

Subsequent processing for protein identification was done through ProteinScape 2.1 (Bruker) interface connected to Mascot Server 2.3 (Matrix Science). Fixed carbamidomethyl cysteine, variable oxidized methionine, single trypsin miscleavage, and appropriate mass tolerances (40 ppm for precursor ions and 0.4 Da for fragments) were specified. Queries were done against *Homo sapiens* proteins downloaded from UniProt^1^ in April 2014, containing 69,060 sequences. Protein identifications were accepted if the ion scores of at least two different matched peptides were higher than the probability of identity threshold (30 at *P* < 0.05). The mass spectrometry proteomics data have been deposited to the ProteomeXchange Consortium via the PRIDE partner repository (Vizcaino et al., 2016) with the dataset identifier PXD005205 and doi: 10.6019/PXD005205.

### Bioinformatics Analysis

Gene Ontology annotation was acquired from the gene ontology project using the PANTHER database for biological procedures and molecular function^2^. Functional enrichment analysis was performed by Fisher’s exact test with False Discovery (FDR) correction, with FDR *P* < 0.05 considered significant (Mi et al., 2016). The protein-protein interaction network was analyzed using the STRING 11.0 database with default settings, while the interaction score was set to medium confidence (0.400)^3^ (Jensen et al., 2009).

### Western Blot Analysis

Equal amounts of proteins from untreated mock-infected and LCMV-infected cells or cells treated with 10 mM N-acetyl-L-cysteine (NAC) for 24 h were separated in 10% SDS-polyacrylamide gel, transferred onto a polyvinylidene difluoride membrane (Immobilon-P; Millipore), and probed with antibodies specific for LCMV NP [in-house generated mAb M87 (Tomaskova et al., 2011)], alpha-enolase (Abcam #ab85086, 1:1000 dilution), GP96/HSP90-beta 1 (Millipore #ABF306, 1:5000 dilution), PRDX4 (Invitrogen #PA3-753, 1:1000 dilution), Phospho-Akt (Ser473) (Cell Signaling Technology #9271, 1:1000 dilution), Akt (Cell Signaling Technology #4691, 1:2000 dilution), β-actin (Cell Signaling Technology #3700, 1:5000 dilution), and alpha-tubulin (Abcam [ab7291], 1:10,000 dilution). Detection was performed with HRP- or IRDye-labeled secondary antibodies visualized with enhanced chemiluminescence or Odyssey CLx imager system, respectively. Densitometric analysis of protein abundance was performed using ImageJ software (Schindelin et al., 2015) or Image Studio Lite software, respectively.

### Measurement of Reactive Oxygen Species Production

The generation of reactive oxygen species (ROS) was assessed using the fluoroprobe 5-(and-6)-chloromethyl-2′,7′-dichlorodihydrofluorescein diacetate (CM-H2DCFDA). Cells (4 × 10^4^ cells/well) cultured in 96-well plates were washed with phosphate-buffered saline supplemented with calcium/magnesium (PBS) and incubated with 10 μM CM-H2DCFDA (Molecular Probes) in PBS for 20 min in the dark at 37°C. After replacement of the reactive agents with PBS, 2′,7′-dichlorofluorescein (DCF) fluorescence was measured at an excitation wavelength of 492 nm and an emission wavelength of 527 nm using the SYNERGY/H4 microplate reader (BioTec Instruments). Values were corrected for background auto-fluorescence of non-stained cells and normalized to the number of cells assessed by DAPI staining. Briefly, at the end of the experiment, cells were fixed with ice-cold methanol for 5 min at −20°C, washed twice with PBS and then incubated with DAPI (1 μg/ml) for 5 min in the dark. After a subsequent washing step with PBS, DAPI fluorescence was measured in each well using 358 nm excitation and 460 nm emission wavelengths.

### Telomere Restriction Fragment (TRF) Analysis

The genomic DNA (gDNA) was isolated from HeLa and HeLa-MX cells using QIAamp DNA Mini Kit (QIAGEN) according to manufacturer’s instructions. Three μg of genomic DNA were digested using a mixture of restriction enzymes (*Hha*I, HinF1, *Msp*I, *Hae*III, *Rsa*I, *Alu*I) as described by Mender and Shay (2015). The DNA fragments were separated in 1% agarose gel for 16 h at 1.6 V/cm and stained with 0.5 μg/mL ethidium bromide solution for 20 min (stained gel served as a loading control). The gel was then incubated for 40 min in denaturation solution (1.5 M NaCl, 0.5 M NaOH), 30 min in neutralization solution (1.5 M NaCl, 0.5 M Tris, pH 7.4) and 30 min in 20× SSC (3 M NaCl, 0.3 M Na-citrate, pH 7.0). The DNA was then transferred to Immobilon NY + membrane (EMD Millipore) with a VacuGene XL blotter (GE Healthcare) in 20× SSC and fixed by incubating the membrane at 80°C for 1 h. The membrane was pre-hybridized for 20 min at 42°C in hybridization buffer (6× SSC, 5× Denhardt solution, 2.5% SDS) and hybridized at 42°C overnight in the same buffer containing radioactively labeled telomere-specific C-rich probe prepared as described by Mender and Shay (2015). The membrane was washed once with wash buffer I (2× SSC, 0.1% SDS) for 15 min at 37°C, twice for 15 min in wash buffer II (0.5× SSC, 0.1% SDS) at 37°C and twice for 15 min at room temperature with wash buffer III (0.5× SSC, 1% SDS). The signal was detected by Personal Molecular Imager FX (BioRad).

### Luciferase Reporter Assay

HeLa and HeLa-MX cells were seeded into a 6-well plate at 4 × 10^5^ cells per well and transfected with 2 μg of the luciferase vector (HRE-luc) containing hypoxia-responsive elements the next day, using the Turbofect Transfection reagent (Thermo Fisher Scientific) according to the manufacturer’s instructions. Cells were co-transfected with 100 ng of pRL-TK Renilla vector (Promega) to normalize for the transfection efficiency. Luciferase reporter construct containing three hypoxia response elements (HRE; 24-mers) from the Pgk-1 gene was a gift from Navdeep Chandel (Emerling et al., 2008) (Addgene plasmid #26731^4^; RRID:Addgene_26731). Reporter gene expression was assessed 48 h after transfection using the Dual Luciferase Reporter Assay System (Promega) and the Synergy HT reader with Gen5 software (BioTec Instruments). Luciferase activity was normalized against Renilla activity.

### Statistical Analyses

Statistical analyses were performed using GraphPad Prism 8 software for Windows, unless stated otherwise. Two-tailed unpaired *T*-test was used to analyze significant differences between two cell groups and one-way ANOVA was used to determine statistically significant differences between three or more independent groups, with *P* < 0.05 considered significant.

## Results

### 2D-DIGE Determines Proteins Differentially Abundant Upon Persistent LCMV Infection

To investigate global protein changes in HeLa cells during persistent infection with the MX strain of LCMV, equal amounts of total proteins prepared from mock-infected and LCMV-infected HeLa cells were subjected to 2D-DIGE analysis. We used three independent biological replicates to generate comprehensive and reliable data (Supplementary Figure S1). The separation strength was satisfactory; the average yield per gel comprised approximately 2600 protein spots. Initial between-gel matching was carried out by manual landmarking, followed by automatic matching and extensive manual evaluation of the matched spots between individual gels in the DeCyder 2D 7.2 Software. Only protein spots showing significance (*P* < 0.05) and at least 1.5-fold difference in abundance were considered as up- or down-regulated. According to our analyses, the abundance of 34 protein spots was significantly altered after LCMV infection. Since DeCyder does not allow identification of proteins present only in one condition and absent in the other, we complemented gel analysis with a manual qualitative study of protein presence between individual samples. In total, 19 protein spots were present only in one condition (mock-infected or LCMV-infected). These 53 protein spots were marked as “proteins of interest (POIs)” and matched against the SYPRO Ruby-stained preparative gel.

### Identification of Significantly Changed Proteins in LCMV-Infected Cells and Their Biological Relevance

**FIGURE 1:**
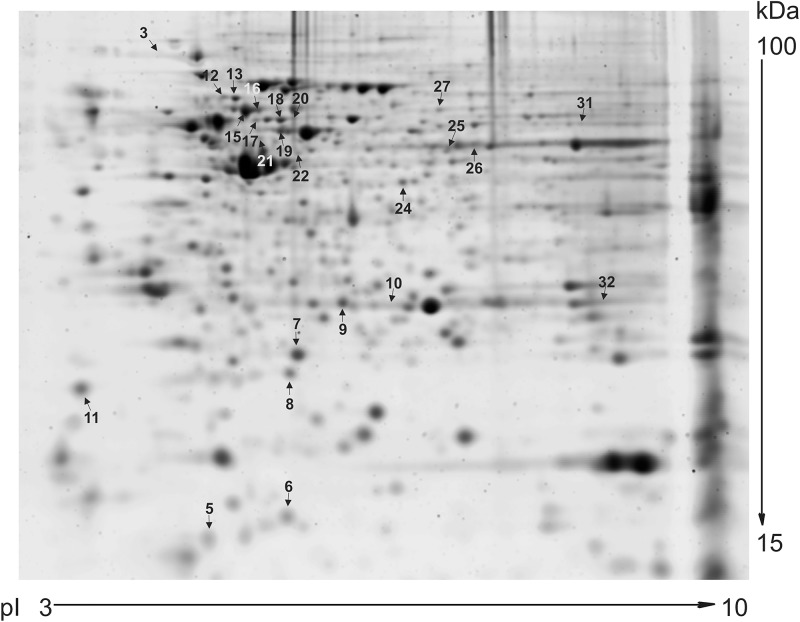
Preparative 2D gel. A total of 100 μg proteins from each sample of LCMV-infected and mock-infected HeLa cells were resolved by 2D PAGE. The protein spots were visualized by SYPRO Ruby Protein Gel Stain. This preparative 2D-gel was used to cut out protein spots that were differentially abundant in analytical gels. Arrows indicate the isolated and identified protein spots with at least 1.5-fold up-regulation or down-regulation. Spots are numbered according to Table 1.

The 30 proteins of interest, which we were able to map on the preparative gel, were excised and analyzed by MALDI-TOF tandem mass spectrometry. Twenty-four protein spots with 21 non-redundant proteins were successfully identified, including 12 up-regulated and 12 down-regulated proteins (Figure 1).

Detailed information on the identified proteins is provided in Table 1. Several proteins were present in more than one spot, namely: heterogeneous nuclear ribonucleoprotein K (hnRNP K; spot 12 and 13, both more abundant upon infection), mitochondrial 60 kDa heat shock protein (hsp60; spot 15 and 16, both less abundant in cells affected by persistent virus), and keratin, type II cytoskeletal 8 (CK-8; spot 19 and 20, which showed opposite changes). These proteins are most likely different post-translational or alternatively spliced variants. Proteins present only in one condition were: (a) unique upon LCMV infection – mitochondrial hydroxymethylglutaryl-CoA synthase, guanine deaminase, annexin A7, and RAB GDP dissociation inhibitor beta; (b) unique in the mock-infected cell line – cellular retinoic acid binding protein 2, prostaglandin E synthase 3, and eukaryotic initiation factor 4A-I. Apart from proteins discussed in more detail below, other accumulated proteins upon infection included galectin 1 and macrophage-capping protein, and less abundant endoplasmin, GTP-binding nuclear protein RAN, peptidyl-prolyl *cis*-*trans* isomerase FKBP4, and adenine phosphoribosyltransferase.

**TABLE 1:** Proteins differentially abundant in LCMV-infected HeLa cells identified by MALDI-TOF tandem mass spectrometry.

**Spot ID**	**UniProt ID**	**Gene name**	**Protein name**	**Mascot score**	**Number of peptides/SC (%)**	**Av. ratio HeLa-MX/HeLa**
3	P14625	HSP90B1	Endoplasmin	133.6	4/4.5	−2.09
5	P09382	LGALS1	Galectin-1	334.8	4/34.8	1.79
6	P29373	CRABP2	Cellular retinoic acid-binding protein 2	255.2	4/30.4	Only HeLa
7	P32119	PRDX2	Peroxiredoxin-2	511.0	9/31.8	−1.5
8	P07741	APRT	Adenine phosphoribosyltransferase	489.7	7/38.9	−1.82
9	Q13162	PRDX4	Peroxiredoxin-4	559.9	9/29.2	−2.08
10	P30041	PRDX6	Peroxiredoxin-6	209.1	5/21.9	−1.71
11	Q15185	PTGES3	Prostaglandin E synthase 3	381.9	7/20.0	Only HeLa
12	P61978	HNRNPK	Heterogeneous nuclear ribonucleoprotein K	201.8	3/7.1	2.41
13	P61978	HNRNPK	Heterogeneous nuclear ribonucleoprotein K	88.7	4/8.4	1.7
15	P10809	HSPD1	60 kDa heat shock protein, mitochondrial	617.7	12/22.7	−2.32
16	P10809	HSPD1	60 kDa heat shock protein, mitochondrial	162.0	5/8.7	−2.08
17	Q01581	HMGCS1	Hydroxymethylglutaryl-CoA synthase	92.9	2/4.4	Only HeLa-MX
18	Q02790	FKBP4	Peptidyl-prolyl *cis-trans* isomerase FKBP4	311.7	8/17.0	−1.61
19	P05787	KRT8	Keratin, type II cytoskeletal 8	218.6	4/9.5	3.07
20	P05787	KRT8	Keratin, type II cytoskeletal 8	247.5	7/12.2	−1.76
21	P60842	EIF4A1	Eukaryotic initiation factor 4A-I	163.0	5/12.8	Only HeLa
22	Q9Y2T3	GDA	Guanine deaminase	67.8	2/4.6	Only HeLa-MX
24	P40121	CAPG	Macrophage-capping protein	122.9	3/11.5	1.62
25	P20073	ANXA7	Annexin A7	98.9	4/4.5	Only HeLa-MX
26	P50395	GDI2	Rab GDP dissociation inhibitor beta	320.7	6/17.3	Only HeLa-MX
27	B7Z2S5	TXNRD1	Thioredoxin reductase 1, cytoplasmic	65.1	2/4.6	1.61
31	P06733	ENO1	Alpha-enolase	158.2	4/16.1	1.92
32	P62826	RAN	GTP-binding nuclear protein Ran	217.1	4/15.7	−1.54

*Positive ratios indicate proteins accumulated in virus-infected cells, negative ratios show proteins more abundant in mock-inoculated controls. SC, sequence coverage.*

**FIGURE 2:**
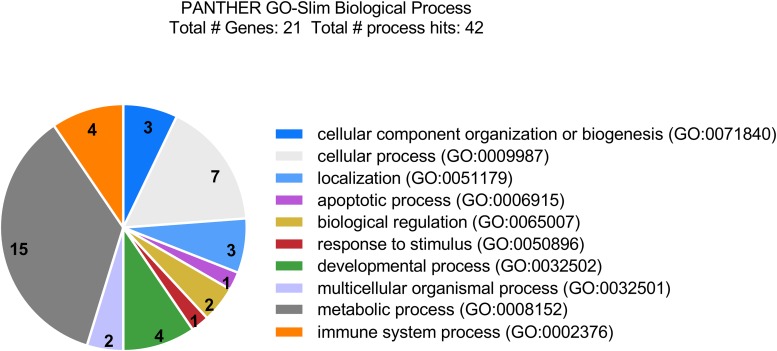
Categorization of differentially accumulated proteins in LCMV-infected HeLa cells by biological functions according to the Gene Ontology database, using PANTHER Classification System. Most of the identified proteins are involved in metabolic processes, cellular processes, such as cellular communication and cell cycle; as well as in immune system processes, and developmental processes.

To better understand the implications of the cellular response to LCMV infection, the corresponding biological functions of the differentially regulated proteins were categorized according to the Gene Ontology database, using the PANTHER Classification System. Most of the proteins with significantly altered abundance were involved in metabolic processes (71.4%; 15 proteins), particularly lipid and glucose metabolism; cellular processes, such as cellular communication and cell cycle (33.3%; 7 proteins); immune system processes (19.0%; 4 proteins); developmental processes (19.0%; 4 proteins); localization (14.3%; 3 proteins); and cellular component organization or biogenesis (14.3%; 3 proteins) (Figure 2). Functional enrichment analysis revealed that the most overrepresented were proteins with peroxiredoxin activity (GO: 0051920; raw *P*-value = 1.03E-07; FDR = 4.81E-04) and proteins that play a role in RNA binding (GO: 0003723; raw *P*-value = 4.98E-07; FDR = 1.16E-03).

**FIGURE 3:**
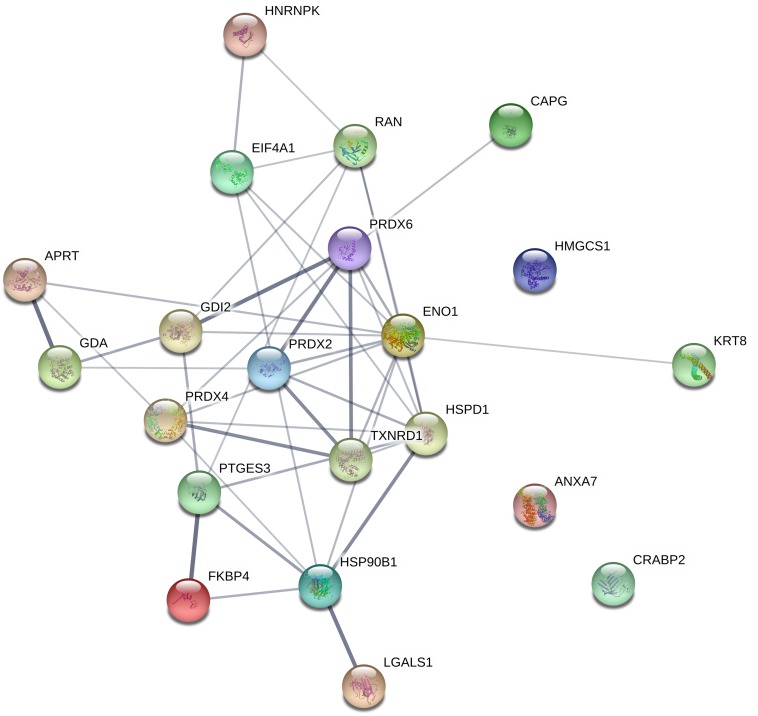
Interaction between differentially regulated proteins in LCMV-infected HeLa cells. The protein network analysis was performed using String 11.0 database. The connecting lines indicate the interactions between proteins. The thickness of the connecting line represents the strength of the association. The interactions network is significantly enriched (*P*-value: <1.0e-16). The protein symbols used in this network analysis are listed in Table 1 (Gene name).

Furthermore, network analysis was conducted to reveal protein-protein interactions. Forty-two associations were observed in the protein network analysis generated by String 11.0 database. The network had significantly more interactions than expected randomly (six expected interactions; PPI enrichment *P*-value < 1.0e-16). Such an enrichment indicates that the proteins are at least partially biologically connected. The confidence view of the protein-protein associations is shown in Figure 3, where the strength of the association is indicated by the thickness of the connecting line (confidence score >0.4). Similar to gene ontology analysis, the most connected proteins have important functions in antioxidant activity, protein folding, and RNA binding.

### Validation of Proteomic Data by Western Blot Analysis

**FIGURE 4:**
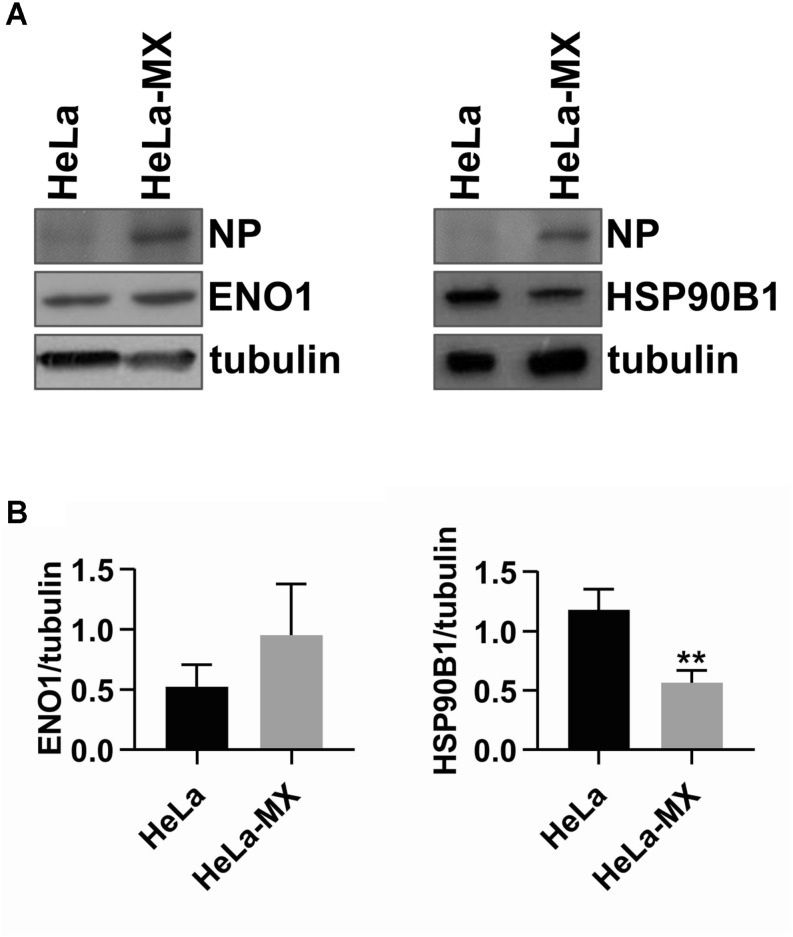
Confirmation of two differentially abundant host proteins in LCMV- and mock-infected HeLa cells by Western blot analysis. **(A)** Immunoblot analysis of ENO1 (left) and HSP90B1 (right) with specific antibodies using whole-cell extracts prepared from LCMV-infected HeLa cells (HeLa-MX) and mock-infected HeLa cells (HeLa). The signal obtained with anti-alpha-tubulin antibody was used as loading control. Presence of NP confirms LCMV infection. One representative of three biological replicates is shown. **(B)** Densitometric analysis of protein abundance was performed using ImageJ software. Relative quantity of proteins was calculated as the ratio of the intensity of each signal to the intensity of the related tubulin internal standard. Values represent the means of three biological replicates. Error bars denote the standard deviations. ^∗∗^*P* < 0.01 (HeLa-MX vs. HeLa).

To validate the changes in protein accumulation patterns in response to LCMV infection, two identified proteins, alpha-enolase (ENO1; more abundant) and heat shock protein 90 kDa beta, member 1 [HSP90B1 (endoplasmin); less abundant] were selected for Western blot analysis. The reason to select HSP90B1 was its important functionality, pinpointed by the String network analysis. Figure 4A shows a prominent although not statistically significant increase in protein abundance of ENO1 and a decrease for HSP90B1 in infected cells. These results are consistent with those observed in the 2D-DIGE analysis. The densitometric analysis of three biological replicates revealed HeLa-MX/HeLa average ratio of 1.8 for ENO1 and −2.1 for HSP90B1, while alpha-tubulin was used as a loading control (Figure 4B). The results were very close to the changes observed in the 2D-DIGE analysis, which were 1.9 for ENO1 and −2.1 for HSP90B1.

### LCMV Infection Results in Increased ROS Production

**FIGURE 5:**
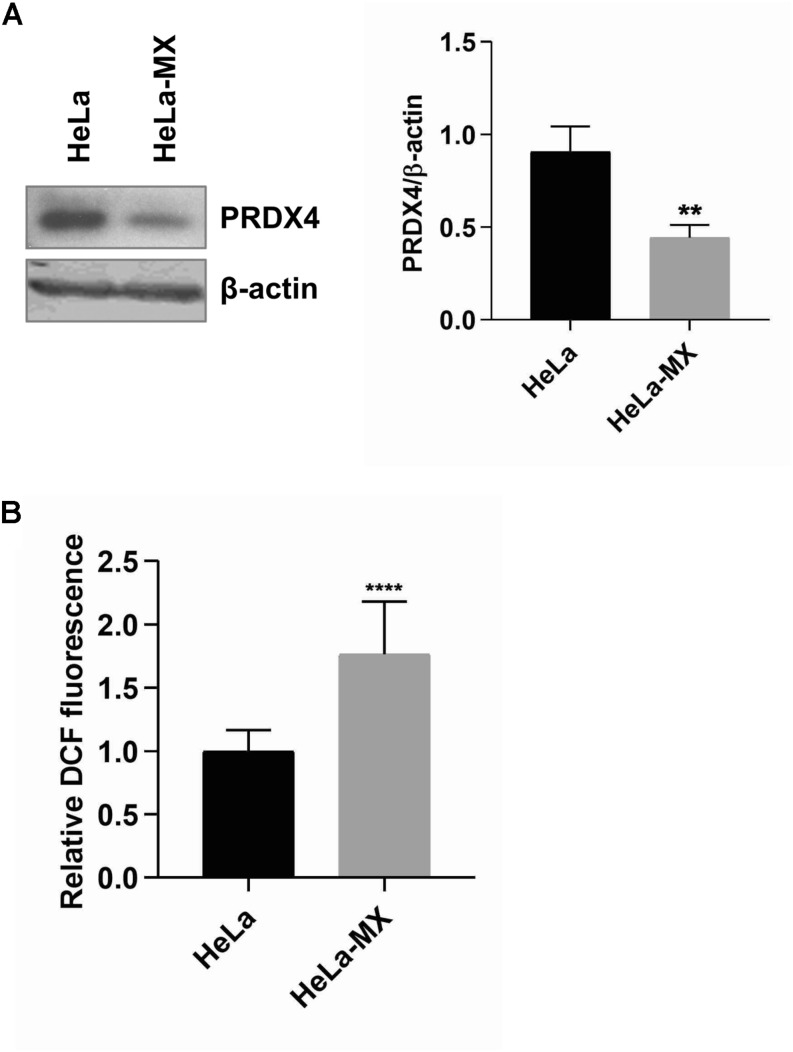
ROS production in LCMV- and mock-infected HeLa cells. **(A)** Immunoblot analysis of PRDX4 (left) with specific antibody using whole-cell extracts prepared from LCMV-infected HeLa cells (HeLa-MX) and mock-infected HeLa cells (HeLa). The signal obtained with anti-β-actin antibody was used as loading control. One representative of three biological replicates is shown. Densitometric analysis of protein abundance (right) was performed using ImageJ software. Relative quantity of proteins was calculated as the ratio of the intensity of each signal to the intensity of the related β-actin internal standard. Values represent the means of three biological replicates. Error bars denote standard deviations. ^∗∗^*P* < 0.01 (HeLa-MX vs. HeLa). **(B)** ROS generation was assessed in a microplate reader using DCF fluorescence and normalized to the number of cells measured by DAPI staining. The results represent the mean from three independent biological experiments, each done in eight replicates. Error bars denote standard deviations. Data are presented as relative increase compared to control (HeLa cells), which was set to 1. ^****^P < 0.0001 (HeLa-MX vs. HeLa).

In this study, we observed a decreased amount of peroxiredoxin 2 (PRDX2), peroxiredoxin 4 (PRDX4), and peroxiredoxin 6 (PRDX6) upon LCMV infection. Peroxiredoxins (Prxs) are members of an ubiquitous family of thiol-specific peroxidases that reduce mainly hydrogen peroxide (H_2_O_2_) to water with the use of reducing equivalents provided through the thioredoxin system (Wood et al., 2003). To confirm the alteration in the abundance of peroxiredoxins during LCMV infection, we selected the most affected PRDX4 for Western blot analysis. Figure 5A shows an almost identical decrease in the expression of PRDX4 (−2.05) in infected cells as we observed in the 2D-DIGE analysis (−2.08).

Since one of the major functions of Prxs is cellular protection against oxidative stress, we hypothesized that decreased levels of these antioxidant proteins may lead to an elevation of the ROS content in infected cells. The measurement of ROS generation in mock- and LCMV-infected cells supported our prediction. We revealed significantly higher production of ROS (1.76-fold) in LCMV-infected cells than in mock-infected cells (Figure 5B). Interestingly, another antioxidant enzyme, thioredoxin reductase 1 (TrxR1), was more abundant in infected cells (Table 1), yet this seems to be insufficient to counterbalance the decrease in the levels of peroxiredoxins.

In order to determine whether the increased ROS level described above is unique to the MX-infected HeLa cells or can also be seen in other cell lines, we decided to use A549 lung epithelial cells, which are commonly used as an *in vitro* model system for arenavirus infections. Similar to HeLa cells, we detected a 1.55-fold induction of ROS content in A549 cells persistently infected with LCMV-MX compared to control cells (Supplementary Figure S2A).

### LCMV Infection Affects the Length of Host Telomeres

Further, we hypothesized that increased levels of ROS may have an effect on DNA of the infected cells. Telomeric sequences composed of arrays of 5′-TTAGGG-3′ repeats are particularly vulnerable to oxidative damage due to stretches of G residues that are susceptible to the conversion to 8-oxoguanine (8-oxoG). Accumulation of 8-oxoG in telomeric repeats may affect accessibility of chromosomal ends to telomerase, binding efficiency of telomere-binding proteins, and/or formation of protective secondary structures such as telomeric loops (von Zglinicki, 2002; Ahmed and Lingner, 2018). As a result, increased levels of ROS yield changes in the length of telomeric tracts.

**FIGURE 6:**
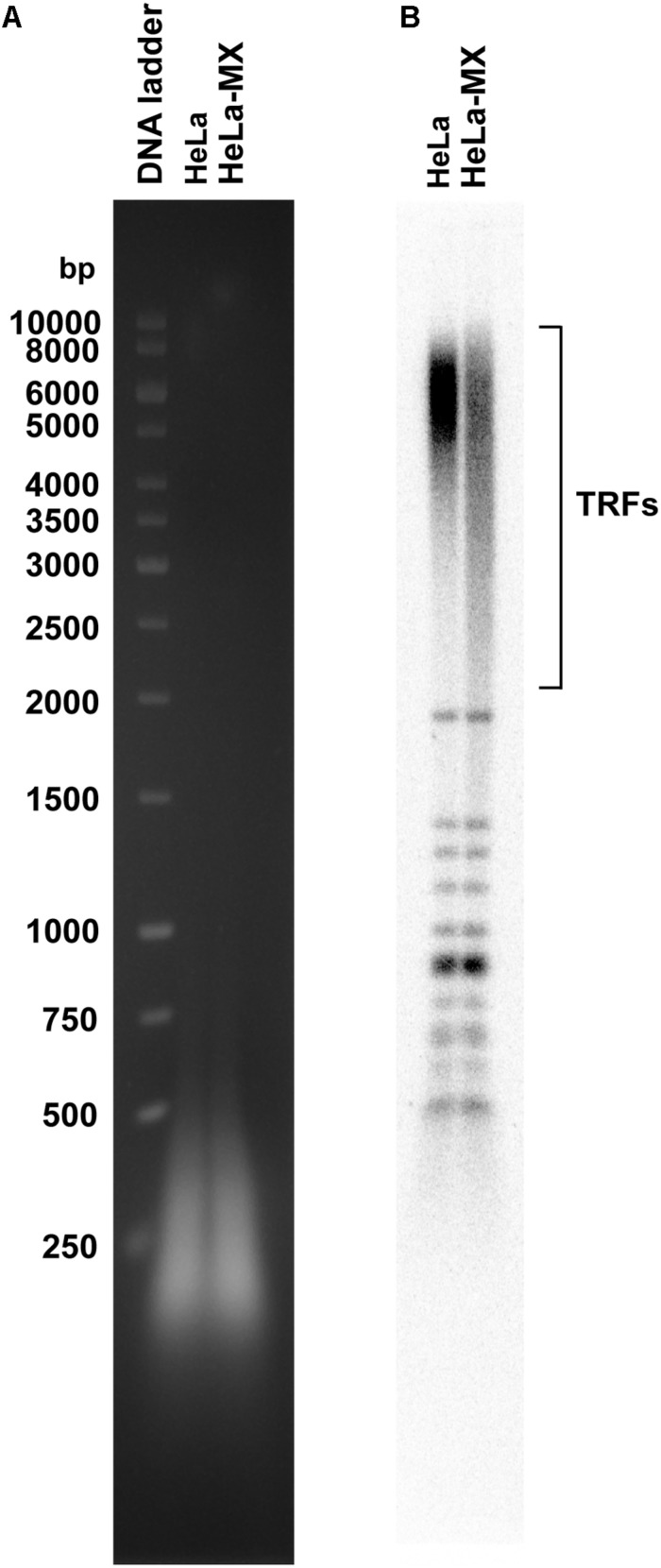
LMCV-infected cells exhibit changes in the pattern of telomeric restriction fragments. **(A)** Total DNA was isolated from uninfected (HeLa) and infected (HeLa-MX) cells, digested by a cocktail of restriction enzymes and the resulting DNA fragments were separated by 1% agarose gel electrophoresis. **(B)** DNA was transferred to nylon membrane and hybridized with a radiolabeled telomeric probe. TRFs (telomeric restriction fragments), DNA ladder (GeneRuler 1 kb DNA Ladder (Thermo Scientific). A representative of three independent experiments is shown.

To test this hypothesis, we isolated genomic DNA from control and infected cells and measured the length of telomere restriction fragments (TRFs). We observed that the infected cells contained a more heterogeneous population of TRFs and a higher proportion of shorter fragments compared with the control cells (Figure 6). This indicates that the infected cells have a compromised ability to maintain the standard length of telomeres.

### LCMV Activates Hypoxia-Inducible Transcription Factor-1 (HIF-1) and Phosphatidylinositol 3-Kinase (PI3K)/Akt Signaling Pathways

**FIGURE 7:**
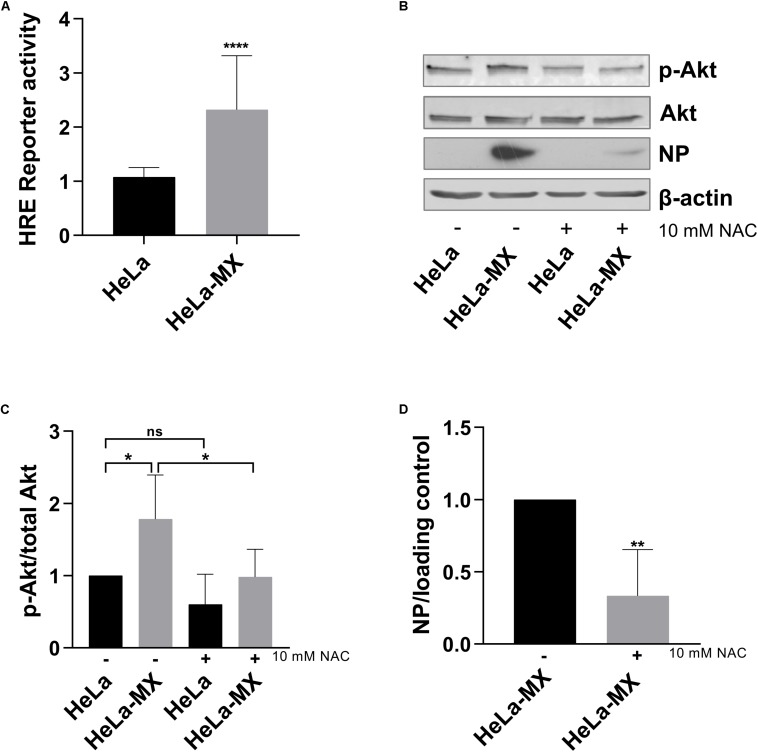
Effect of LCMV infection on HIF-1 and PI3K/Akt activation. **(A)** Mock- and LCMV-infected HeLa cells were transfected with HRE-Luc, and the reporter protein (luciferase) activity was determined at 48 h post-transfection. Reporter protein activity was normalized to uninfected, control-transfected cells. Values represent means of three independent experiments, each done in eight replicates. Error bars denote standard deviations. Data are presented as a relative increase compared to control (HeLa cells), which was set to 1. ^****^P < 0.0001 (HeLa-MX vs. HeLa). **(B)** Immunoblot analysis of p-Akt (S473), total Akt, and viral NP with specific antibodies using whole-cell extracts prepared from untreated (−) mock-infected HeLa cells (HeLa) and LCMV-infected HeLa cells (HeLa-MX) or cells treated (+) with 10 mM NAC for 24 h. The signal obtained with anti-β-actin antibody was used as loading control. One representative of at least four biological replicates is shown. **(C)** Densitometric analysis of protein abundance was performed using ImageJ software or Image Studio Lite software. Relative quantity of p-Akt was calculated as the ratio of the intensity of each signal to the intensity of the related signal for total Akt. Values represent the means of six biological replicates. Error bars denote the standard deviations. Data are presented as relative change compared to control (untreated HeLa cells), which was set to 1. ^∗^*P* = 0.0119 (HeLa vs. HeLa-MX), ^∗^*P* = 0.0101 (HeLa-MX vs. HeLa-MX NAC), *P* = 0.3002 (HeLa vs. HeLa NAC). **(D)** Densitometric analysis of protein abundance was performed using ImageJ software or Image Studio Lite software. Relative quantity of NP was calculated as the ratio of the intensity of each signal to the intensity of the related β-actin or tubulin internal standard, respectively. Values represent the means of four biological replicates. Error bars denote the standard deviations. Data are presented as relative change compared to untreated HeLa-MX cells, which was set to 1. ^∗∗^*P* = 0.0059 (HeLa-MX NAC vs. HeLa-MX).

It has become apparent in recent decades that less-reactive ROS, especially hydrogen peroxide, can function as intracellular signaling molecules regulating multiple physiological and pathological cell processes. It has been shown that ROS mediate activation of several signaling pathways and transcription factors, including the PI3K/Akt signaling cascade (Lee et al., 1998, 2002) and HIF (Patten et al., 2010). To assess biological implications of increased ROS in infected cells, we investigated whether LCMV infection affected transcriptional activity of HIF-1. Using a luciferase reporter containing HRE, we detected a modest, but significant increase in HIF-1 transactivation in infected HeLa cells in comparison to control cells (Figure 7A). Further, we evaluated activation of Akt by probing cellular protein lysates for phospho-Akt (S473), which is a marker for activated Akt. As shown in Figures 7B,C, phosphorylation of Akt was induced in infected cells, while overall Akt levels were similar in both infected and uninfected cells.

Since we assumed that activation of Akt was triggered by redox signaling, we further investigated the effect of antioxidants on infected cells. We detected decreased levels of phospho-Akt in LCMV-infected HeLa cells treated with N-acetyl cysteine. Moreover, inhibition of ROS accumulation by the antioxidant also decreased the levels of viral NP (Figures 7B,D). In addition, we observed similar impact of antioxidant treatment in LCMV-infected A549 cells, although the reduction in NP levels was less pronounced (Supplementary Figure S2B).

These data suggest that LCMV maintains its replication in persistently infected cells by regulating redox signaling through modulation of local levels of H_2_O_2_ and subsequent activation of cellular processes that it uses for its own benefit.

## Discussion

Viruses and host cells have established complex, dynamic interactions that either facilitate the efficiency of viral infection or defend the host against invading pathogens. These interactions are crucial for the regulation of metabolism and other processes in the host cell and their elucidation is critical for a detailed description of mechanisms of viral pathogenesis, eventually leading to effective treatment.

In this report, we investigated the host response to persistent LCMV infection using 2D-DIGE-based proteomic approach. As the model system of persistent infection we used human Hela cells long-term infected with LCMV MX isolate (over 50 passages). The MX isolate likely represents a LCMV variant that has undergone complex adaptations. It is characterized by reduced accumulation of viral glycoproteins on the surface of infected cells and by spreading to uninfected cells mainly through cell-cell contact without the release of infectious viral particles, all of which strongly resembles persistent LCMV neuronal infection in its native host (Rodriguez et al., 1983). The use of HeLa-MX cells has several advantages, such as the feasibility of use of a large number of cells for proteomic analysis, reproducibility, and viral persistence resembling a life-long chronic infection. However, in terms of natural LCMV infection, HeLa cells possess some limitations and one needs to be cautious about generalizing the results. Therefore, we performed some of the experiments also on the A549 cell line, a model of type II alveolar epithelial cells (Supplementary Figure S2). This cell type is the first to be in contact with the virus during primary infection. It has also been shown that A549 cells are capable of antigen presentation (Salik et al., 1999). Although the data demonstrating chronic infection in humans are not available, the persisting and reactivated virus may be a source of unexplained complications (often fatal) in immunosuppressed transplant recipients, as well as in developing embryos (Peters, 2006; Bonthius, 2009). Thus, a better understanding of cellular processes exploited or subverted by viruses during persistent infection can help develop new strategies to treat mammarenavirus infections in humans.

Our analysis revealed 53 protein spots differentially accumulated upon persistent LCMV infection in HeLa cells. 24 proteins were identified with high confidence.

Remarkably, the abundance of several antioxidant enzymes was shown to be significantly altered. Namely, the levels of PRDX2, PRDX4, and PRDX6 were 1.5–2.08 times lower in LCMV-infected cells (Table 1). In addition, functional enrichment analysis, as well as network analysis, pointed out that the antioxidant system was in the center of virus-induced host proteome response (Figure 3). The key functions of Prxs include cellular protection against oxidative stress and modulation of signaling pathways that use hydrogen peroxide as a second messenger (Rhee et al., 2005a, b). A significant amount of published data suggests that Prxs are hydrogen peroxide sensors that play a central role in redox signaling (Rhee et al., 2012; Latimer and Veal, 2016; Netto and Antunes, 2016). This is implicated in a number of biological processes, including growth factor signaling, cell differentiation, and cytokine production (Hampton and O’Connor, 2016). Regulation of Prxs modifies the concentration of H_2_O_2_ and thereby facilitates its signaling functions. We indeed confirmed a slight but significant elevation of the ROS content alongside the downregulation of Prxs by LCMV both in HeLa and A549 cells (Figure 5B and Supplementary Figure S2A).

ROS, in the form of H_2_O_2_, can modify protein function, and/or structure through the mechanism of cysteine oxidation that influences a number of signaling cascades. For example, ROS activate PI3K either directly or by inactivating the phosphatase PTEN via oxidizing cysteine residues within its active site, resulting in an increased activation of Akt and modulation of its downstream targets (Lee et al., 2002; Connor et al., 2005). Once activated, Akt promotes cell survival, growth, metabolism, and proliferation by phosphorylating various effectors (Ersahin et al., 2015). In line with these findings, we observed enhanced levels of active Akt in LCMV-infected cells. In addition, inhibition of ROS by N-acetyl cysteine led to suppressed phosphorylation of Akt in infected cells, suggesting that Akt becomes activated in a ROS-dependent manner (Figures 7B,C). Notably, the treatment with antioxidants also resulted in reduced levels of viral NP in HeLa, as well as in A549 LCMV-infected cells, suggesting a link between ROS-dependent signaling and virus replication (Figures 7B,D and Supplementary Figure S2B). These findings are in accordance with previous studies that have demonstrated that ROS generated in response to LCMV play an important role in virus binding and subsequent virus replication (Michalek et al., 2008). Moreover, it has been shown that the PI3K/Akt pathway plays an important role in different steps of the life cycle of a variety of viruses. For instance, infection with the New World mammarenavirus Junin virus has been shown to induce the PI3K/Akt pathway, and inhibition of this pathway has resulted in a decreased infectious virus yield because of blocking the recycling of the transferrin receptor engaged in JUNV cell entry (Linero and Scolaro, 2009). Furthermore, PI3K/Akt pathway inhibition reduced budding and, to a lower degree, LCMV RNA synthesis, but not cell entry (Urata et al., 2012). Additionally, the SARS coronavirus induced weak activation of Akt that was crucial for the establishment of persistence (Mizutani et al., 2005). In contrast, PI3K/Akt pathway inhibition could not interfere with the establishment of persistent JUNV infection in Vero cells, and this pathway was not crucial for the maintenance of JUNV persistence (Linero and Scolaro, 2009).

Reactive oxygen species also regulate the activation of several transcription factors, including HIFs. HIF-1 is the main hypoxia-inducible transcription factor responsible for cell and tissue adaptation to low oxygen, by controlling cell metabolism, proliferation and survival, erythropoiesis and angiogenesis (Semenza, 2003). There is increasing evidence demonstrating that non-hypoxic stimuli can also activate HIF-1. It has been shown that the PI3K/Akt/mTOR signaling cascade directly increases the expression of HIF-1 encoding gene on the transcriptional and translational levels (Dery et al., 2005). Any aberrant stimulation of this pathway leads to activation of HIF-1α, even in normoxic conditions. An increased ROS production has been reported as essential for increased HIF translation through the PI3K pathway (Iommarini et al., 2017) and for HIF stabilization through the inactivation of prolyl hydroxylases in a non-hypoxic system (Diebold and Chandel, 2016). Accordingly, we demonstrated enhanced HIF-1 transactivation in LCMV-infected cells (Figure 7A), as well as increased expression of ENO1 (Figure 4), a typical HIF-1 target gene (Semenza et al., 1996). Interestingly, HSP90B1 expression was reduced by persistent LCMV (Figure 4), which has also been previously reported in the case of hypoxia in MIN6 cells (Bensellam et al., 2016). PI3K/Akt signaling, as well as the HIF transcription factors, have in common that they play an essential role in regulating cellular metabolism (Dery et al., 2005; Yu and Cui, 2016). In view of that, we observed that a large number of proteins identified in this study, such as the above mentioned alpha-enolase or hydroxymethylglutaryl-CoA synthase, are involved in metabolism, as indicated by the GO analysis (Figure 2).

Moreover, we have shown previously that the exposure of MX-infected HeLa cells to chronic hypoxia resulted in a HIF-dependent increase of viral replication and enhanced formation of infectious virions (Tomaskova et al., 2011). In addition, there is accumulating evidence that many viruses, through various mechanisms, affect the HIF-1 pathway, causing multiple downstream effects, such as modifying host cell metabolism, stimulating inflammation, and facilitating viral replication (Morinet et al., 2013; Cuninghame et al., 2014). Some viruses, such as the vaccinia virus and hepatitis C virus impair HIF-1α prolyl hydroxylation, which leads to its stabilization (Nasimuzzaman et al., 2007; Mazzon et al., 2013). Hepatitis B virus stabilizes HIF-1α by diminishing the interaction between VHL and HIF-1α (Moon et al., 2004). Influenza virus A H1N1 inhibits the proteasome and decreases FIH-1 expression, thereby activating the HIF-1 pathway by stabilizing HIF-1α (Ren et al., 2019).

Contrary to downregulated Prxs, we observed a 1.6-fold increased abundance of cytoplasmic TrxR1 (encoded by *TXNRD1*) in LCMV-infected cells (Table 1). The thioredoxin (Trx) system consisting of NADPH, TrxR, and thioredoxin, is a key antioxidant mechanism in protection against oxidative stress. It regulates protein dithiol/disulfide balance through its disulfide reductase activity (Arner, 2009). As already mentioned, Trx provides electrons to peroxiredoxins to remove reactive oxygen and nitrogen species. Trx reductase then reduces oxidized Trx back using the reducing NADPH equivalents. Thus, Trx reductase plays a crucial role in regeneration of a catalytically active form of Prxs (Lu and Holmgren, 2014). We can, therefore, speculate that elevated levels of TrxR1 can maintain proper accumulation of local H_2_O_2_ to ensure that it acts as a signaling molecule and does not cause oxidative damage. Based on the abovementioned facts, it is possible that LCMV modulates the delicate interplay inside cells between oxidants and antioxidants, and determines the activity profile for a variety of proteins that maintain persistent infection (such as transcription factors, kinases and phosphatases, cytoskeletal proteins, or metabolic enzymes).

On the other hand, elevated ROS levels in infected cells can be a double-edged sword, as they can negatively affect genomic stability of host cells. Telomeres, the nucleo-protein complexes at the chromosomal ends, are essential parts of the genome providing solutions to both end-protection and end-replication problems (Shay and Wright, 2019). The solution to end-protection problem is provided by a specialized protein complex called shelterin (de Lange, 2010), in combination with the formation of a secondary structure called the telomeric loop (Griffith et al., 1999). The end-replication problem is solved by various means. In human germ or stem cells, and most cancer cells (including HeLa), telomeres are preserved by telomerase, a reverse transcriptase using an RNA subunit as a template for extension of an array of 5′-TTAGGG-3′ repeats (Shay and Wright, 2019). Guanine residues in telomeric repeats are particularly susceptible to oxidation resulting in their conversion to 8-oxoG (Oikawa and Kawanishi, 1999). Accumulation of this oxidized purine compromises the telomere maintenance system in a quite complex way, including inhibition of binding of the shelterin components TRF1 and TRF2, the formation of t-loop, and/or the inhibition of telomerase (Ahmed and Lingner, 2018). Thus, increased intracellular levels of ROS often result in telomere shortening (von Zglinicki, 2002), as observed in this study in the case of HeLa cells infected by LCMV (Figure 6). Furthermore, it was shown previously by the Lingner group that telomeres physically interact with PRDX1 and PRDX2 (Aeby et al., 2016). Although the levels of PRDX1 were not substantially affected in HeLa-MX cells (data not shown), PRDX2 exhibited a 1.5-fold decrease (Table 1). As PRDX2 was shown to interact with telomeres in the S phase of the cell cycle (Aeby et al., 2016), reduction in its amount in HeLa cells infected by LCMV may contribute to a detrimental effect of ROS on the maintenance of their telomeric repeats.

Nevertheless, our data suggest that LCMV affects redox signaling and metabolic pathways that support an anabolic program required for efficient virus replication and/or maintenance of persistent infection. The precise mechanism of how LCMV regulates antioxidant-scavenging proteins in order to effectively manage amounts of ROS still remains unknown and requires further investigation.

## Conclusion

Although research on LCMV has led to many insights into viral persistence, the answers to several crucial questions about host-LCMV interactions during persistent infection are still lacking. This study analyzed proteomic changes in a human cell line upon persistent infection with LCMV using quantitative gel-based proteomics. We identified 24 modulated host proteins, which play important roles in a number of cellular processes. Uncovering their roles in persistent LCMV infection can broaden our understanding of the complex and dynamic virus-host cell interactions and be valuable for antiviral research. We provided experimental evidence that LCMV negatively affects the levels of antioxidant enzymes peroxiredoxins leading to elevated intracellular content of ROS. These changes were accompanied by activation of HIF-1 and PI3K/Akt signaling pathways as well as changes in the profile of telomeric restriction fragments. The treatment with antioxidants resulted in reduced level of viral nucleoprotein, indicating that virus replication and ROS-dependent signaling are interconnected processes.

## Data Availability Statement

The datasets generated for this study can be found in the ProteomeXchange Consortium via the PRIDE partner repository, dataset identifier PXD005205.

## Author Contributions

JT and LT conceived and designed the experiments. MB, MD, and JT analyzed the data. JT wrote the first draft of the manuscript. MB, MD, and LT wrote the sections of the manuscript. All authors performed the experiments, contributed to the manuscript revision, read, and approved the submitted version.

## Conflict of Interest

The authors declare that the research was conducted in the absence of any commercial or financial relationships that could be construed as a potential conflict of interest.

## Footnotes

1 http://uniprot.org

2 http://pantherdb.org/

3 http://string-db.org

4 http://n2t.net/addgene:26731

## Supplementary Material

FIGURE S1 2D-DIGE images of biological triplicates. A 50 μg of each sample were labeled with 400 pmol of either Cy3 or Cy5 dye. Preferential binding of the dyes was evaluated using dye-swap technique between individual replicates of the analytical gel. In panels **(A,C)** the HeLa protein sample was labeled with Cy3 dye (green channel) while Cy5 labeling (red channel) was used for the HeLa-MX. Panel **(B)** represents replicate gel No. 2 where the HeLa sample was labeled with Cy5 and the HeLa-MX was labeled with Cy3. Intra- and inter-gel variations were normalized according to the internal standard consisting of all analyzed samples in ratio 1:1 labeled with Cy2 dye. Once normalized, these analytical 2D gels were used to analyze the differential abundance of proteins between HeLa and Hela-MX samples.

FIGURE S2 Reactive oxygen species production and antioxidant treatment in LCMV- and mock-infected A549 cells. **(A)** ROS generation was assessed in a microplate reader using DCF fluorescence and normalized to the number of cells measured by DAPI staining. The results represent the mean from three independent biological experiments, each done in eight replicates. Error bars denote standard deviations. Data are presented as relative increase compared to control (A549 cells), which was set to 1. ^****^*P* < 0.0001 (A549-MX vs. A549). **(B)** Immunoblot analysis of viral NP with specific antibodies using whole-cell extracts prepared from untreated (−) mock-infected A549 cells (A549) and LCMV-infected A549 cells (A549-MX) or cells treated (+) with 10 mM NAC for 24 h. The signal obtained with anti-β-actin antibody was used as loading control. One of two biological replicates is shown.
