# Supplementary figures and images for: Quantitative Proteomics Reveal Peroxiredoxin Perturbation Upon Persistent Lymphocytic Choriomeningitis Virus Infection in Human Cells
Source: Front Microbiol. 2019 Oct 25;10:2438. doi: 10.3389/fmicb.2019.02438 (PMC6823195; doi:10.3389/fmicb.2019.02438)

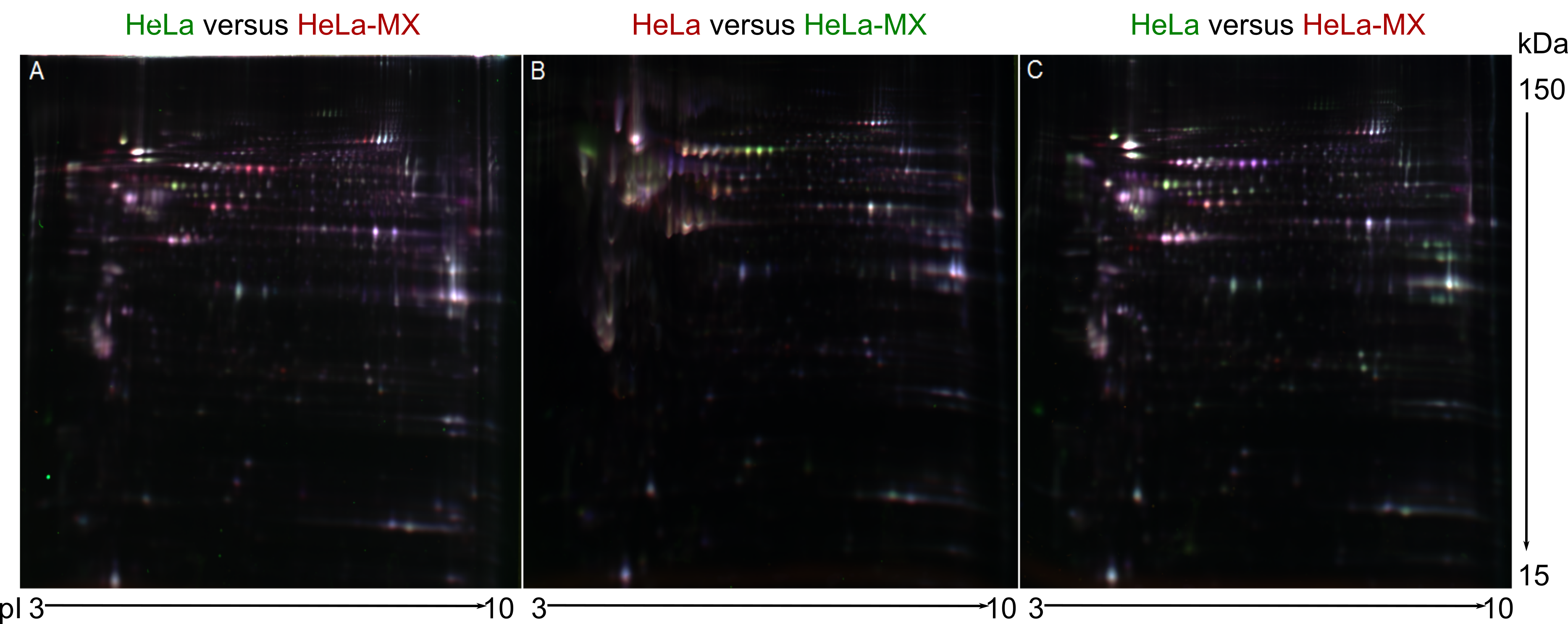

Supplement: FIGURE S1 — 2D-DIGE images of biological triplicates. A 50 μg of each sample were labeled with 400 pmol of either Cy3 or Cy5 dye. Preferential binding of the dyes was evaluated using dye-swap technique between individual replicates of the analytical gel. In panels (A,C) the HeLa protein sample was labeled with Cy3 dye (green channel) while Cy5 labeling (red channel) was used for the HeLa-MX. Panel (B) represents replicate gel No. 2 where the HeLa sample was labeled with Cy5 and the HeLa-MX was labeled with Cy3. Intra- and inter-gel variations were normalized according to the internal standard consisting of all analyzed samples in ratio 1:1 labeled with Cy2 dye. Once normalized, these analytical 2D gels were used to analyze the differential abundance of proteins between HeLa and Hela-MX samples. [file Image_1.TIF]

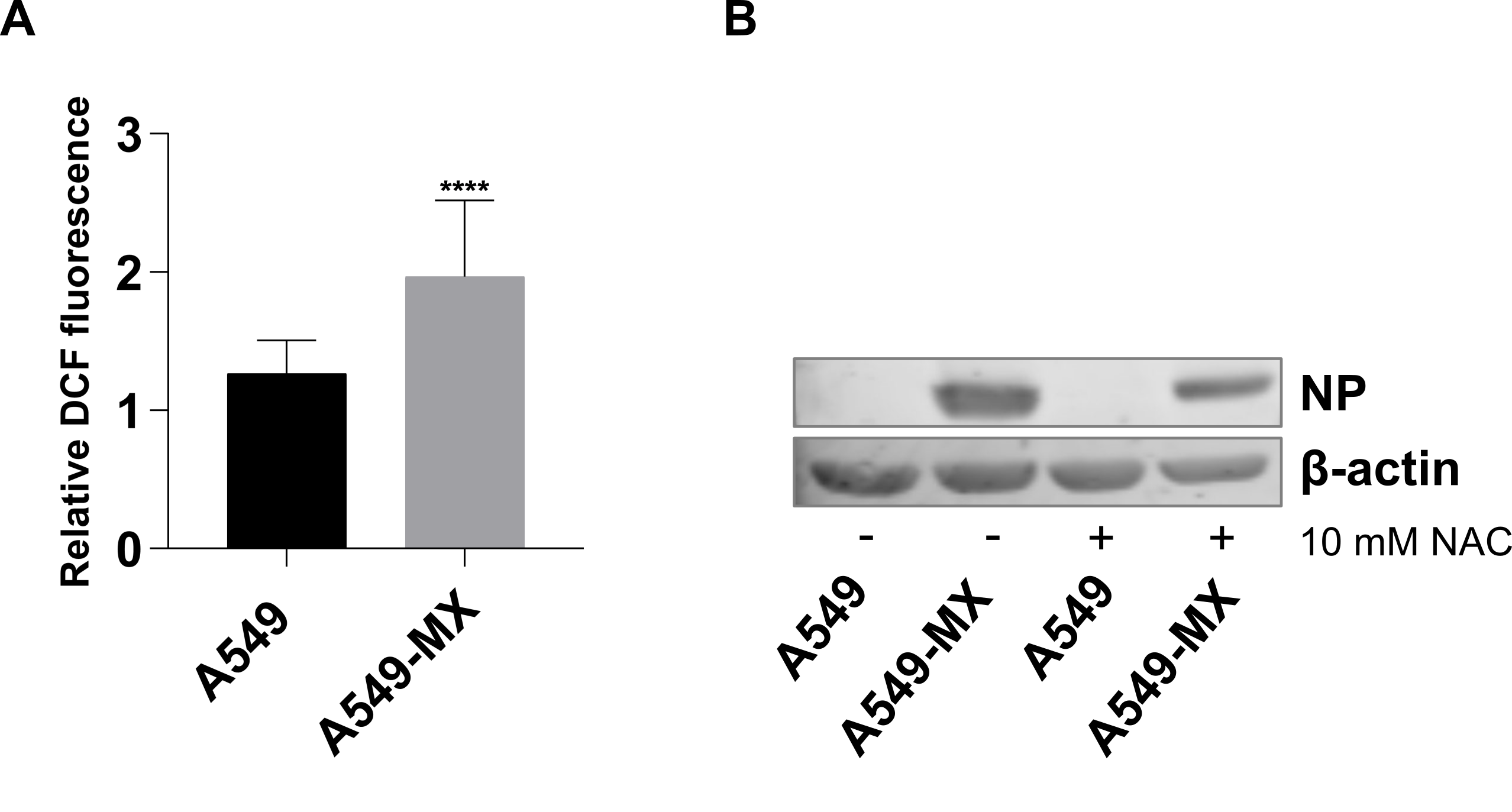

Supplement: FIGURE S2 — Reactive oxygen species production and antioxidant treatment in LCMV- and mock-infected A549 cells. (A) ROS generation was assessed in a microplate reader using DCF fluorescence and normalized to the number of cells measured by DAPI staining. The results represent the mean from three independent biological experiments, each done in eight replicates. Error bars denote standard deviations. Data are presented as relative increase compared to control (A549 cells), which was set to 1. ****P < 0.0001 (A549-MX vs. A549). (B) Immunoblot analysis of viral NP with specific antibodies using whole-cell extracts prepared from untreated (−) mock-infected A549 cells (A549) and LCMV-infected A549 cells (A549-MX) or cells treated (+) with 10 mM NAC for 24 h. The signal obtained with anti-β-actin antibody was used as loading control. One of two biological replicates is shown. [file Image_2.TIF]
